# Adaptive Correlation Space Adjusted Open-Loop Tracking Approach for Vehicle Positioning with Global Navigation Satellite System in Urban Areas

**DOI:** 10.3390/s150921581

**Published:** 2015-08-28

**Authors:** Hang Ruan, Jian Li, Lei Zhang, Teng Long

**Affiliations:** Key Laboratory of Electronic and Information Technology in Satellite Navigation (Beijing Institute of Technology), Ministry of Education, School of Information and Electronics, Beijing Institute of Technology, No. 5 Zhongguancun South Street, Haidian District, Beijing 100081, China; E-Mails: ruanhang@bit.edu.cn (H.R.); aerolong@bit.edu.cn (L.Z.); longteng@bit.edu.cn (T.L.)

**Keywords:** GNSS, vehicle positioning, open-loop tracking, pseudo range accuracy

## Abstract

For vehicle positioning with Global Navigation Satellite System (GNSS) in urban areas, open-loop tracking shows better performance because of its high sensitivity and superior robustness against multipath. However, no previous study has focused on the effects of the code search grid size on the code phase measurement accuracy of open-loop tracking. Traditional open-loop tracking methods are performed by the batch correlators with fixed correlation space. The code search grid size, which is the correlation space, is a constant empirical value and the code phase measuring accuracy will be largely degraded due to the improper grid size, especially when the signal carrier-to-noise density ratio ($C/N_{0}$) varies. In this study, the Adaptive Correlation Space Adjusted Open-Loop Tracking Approach (ACSA-OLTA) is proposed to improve the code phase measurement dependent pseudo range accuracy. In ACSA-OLTA, the correlation space is adjusted according to the signal $C/N_{0}$. The novel Equivalent Weighted Pseudo Range Error (EWPRE) is raised to obtain the optimal code search grid sizes for different $C/N_{0}$. The code phase measuring errors of different measurement calculation methods are analyzed for the first time. The measurement calculation strategy of ACSA-OLTA is derived from the analysis to further improve the accuracy but reduce the correlator consumption. Performance simulation and real tests confirm that the pseudo range and positioning accuracy of ASCA-OLTA are better than the traditional open-loop tracking methods in the usual scenarios of urban area.

## 1. Introduction

Vehicle positioning with global navigation satellite system (GNSS) in urban areas is challenging for the reason that satellite signals often suffer from power attenuation and multipath. As the pilot signal is transmitted with the origin data signal in the new GNSS such as the European Galileo and the modernization of the US Global Positioning System (GPS) [1,2,3], the limitation of the coherent integration length brought by the unknown phase inversion no longer exists. As a result, it is possible to further improve the GNSS receiver sensitivity in urban area. However, for the traditional closed-loop tracking approach, the integration length cannot be extensively extended since closed loop is a feedback system and long integration length will break the loop stability. Moreover, it has been verified that lengthening the integration time has little improvement for the sensitivity of the closed-loop tracking [4]. In addition, for multipath mitigation, specially designed methods are also needed.

The block processing based open-loop tracking technique has been proposed to efficiently overcome the limitations of the traditional closed-loop sequential tracking technique for GNSS receivers [5]. The basic process of open loop tracking is detailed in [6]. As it is an open-loop system, open-loop signal tracking is absolutely stable and the power accumulation length can be greatly expanded which will tremendously improve the signal receiving sensitivity [7,8]. Moreover, the sensitivity will be further improved by the introduction of the pilot channel which eliminates the restriction to lengthen the coherent integration length [9]. Additionally, the time-frequency two-dimension correlation result of the open-loop tracking technique makes it convenient to distinguish the line-of-sight (LOS) signal and multipath signal accurately in the harsh urban environments [10,11]. The open-loop tracking method can also detect and lock the signal quickly since it combines the acquisition and tracking stage. As a result, high dynamic signal tracking and limb scan signal tracking can be achieved by it as well [12,13,14].

Hence, the open-loop signal tracking method realized by batch correlators has been applied to vehicle positioning in urban areas [15,16]. In the method, by lengthening the integration time, the sensitivity is improved so that the weak signal in urban area can be detected. With the help of the time-frequency image, the LOS signal is picked out and multipath is mitigated. In order to further keep the LOS signal peak away from multipath peaks, the strategy to choose the most accurate peak instead of the dominate peak among the correlation results is proposed [17]. In [18], amplitude threshold is used to categorize the LOS signal, multipath and noise to keep the correctness of peak detection. The time to position is shortened by the single stage quickly signal locking. However, including the traditional scheme that computes the correlation results with real correlators and the interpolation based correlation calculation method in [6], the pseudo ranges of all the previously designed open loop methods are measured by discrete code search grids with fixed empirical width. No previous study has focused on the impacts of the grid size on the measurement accuracy of open-loop tracking. In fact, the measuring accuracy is probably distorted by the inapposite grid sizes. If the grid is too small, the LOS signal energy that adjacent search grids contain is close to each other so that the most accurate peak is difficult to be distinguished from its adjacent ones and false peak detection will tremendously destroy the accuracy especially when signal power is low. While, for large grid size, it is easy to detect the correct peak but the relatively large grid size brings large uncertainty for the measurement estimation. Although early minus late discriminator (EMLD) is introduced to eliminate the uncertainty [8,19], the measurement accuracy will still be highly degraded when signal power decreases.

In this paper, Adaptive Correlation Space Adjusted Open-Loop Tracking Approach (ACSA-OLTA) is proposed to improve the code phase measurement accuracy of open-loop tracking. In the method, different from the previous open-loop tracking schemes, the space between the batch correlators, namely the code search grid size, is adjusted according to the signal carrier-to-noise density ratio ($C/N_{0}$) which is an indicator of the signal power [20]. The code phase calculating methods are also switched according to $C/N_{0}$. Since the grid size should not be too large or too small so as to gain the balance between the errors induced by the uncertainty within the grid and false peak detection at the same time, the new Equivalent Weighted Pseudo Range Error (EWPRE) is raised based on the false peak detection probability and mean measurement error of the search grids to gain the optimal grid size for different $C/N_{0}$. The code phase measuring error of EMLD is analyzed and compared with other measurement extracting method of open loop tracking for the first time. The $C/N_{0}$ to switch the pseudo range calculation methods is developed by accuracy comparison to further improve the performance but reduce the correlators needed.

The remainder of the paper is organized as follows. In Section 2, the basic concept of open-loop signal tracking method is described based on the batch architecture of GNSS signal processing. Hereafter in Section 3, the ACSA-OLTA is proposed via the calculation of the designed EWPRE and the measurement deviation. Section 4 presents the simulation and the test with the help of the collected real satellite signal data which confirm the performance improvements of ACSA-OLTA. Section 5 concludes the paper.

## 2. Open-Loop Tracking of GNSS Signals

**Figure 1:**
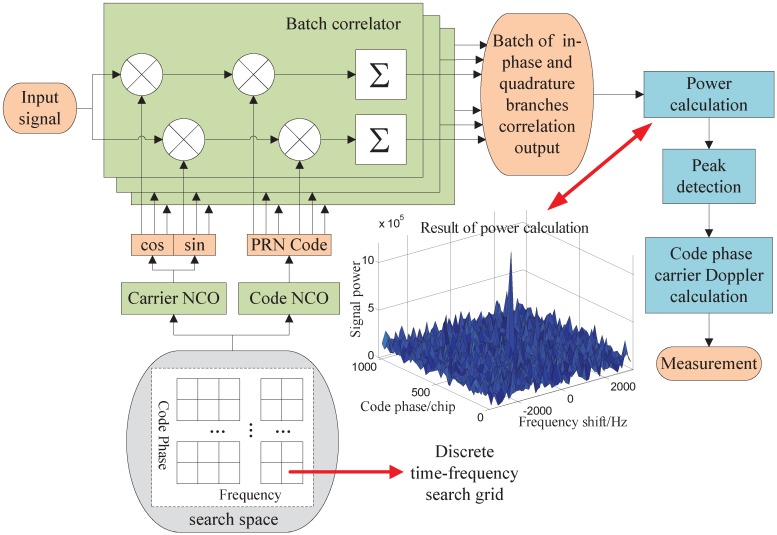
The block diagram of the batch correlators based open-loop tracking method.

This section reviews the main concept of the open-loop tracking of GNSS signals. The block diagram of the batch correlators based open-loop tracking method is illustrated in Figure 1. The input signal is transferred to multiple identical correlators at the same time. In the presence of Additive Gaussian White Noise (AGWN), the input signal is expressed as follow

(1) $$s\left(t_{n}\right)=\sqrt{2P}D\left(t_{n}-\tau \right)c\left(t_{n}-\tau \right)cos\left(2\pi \left(f_{IF}+f_{d}\right)t_{n}+\varphi_{0}\right)+n_{w}\left(t_{n}\right)$$

where $t_{n}$ is the discrete sampling time: $t_{n}=t_{0}+nT_{s}$ and $T_{s}$ is the sampling period. *P* is the incoming signal power. D(·) is the navigation message data or secondary code. c(·) is the Pseudo Random Noise (PRN) primary code. τ is the code phase shift of the incoming signal. $f_{IF}$ is the signal down-conversion frequency. $f_{d}$ is the instantaneous value of the Doppler frequency shift of the received satellite signal. $\varphi_{0}$ is the initial value of the carrier phase. $n_{w}$ is the additive Gaussian white noise with the single band power density of $N_{0}$. Since an orthogonal set of binary pseudorandom codes are generated for different satellites, the signals from different satellites can be processed independently from each other [21]. As a result, the input signal model here contains only one satellite for simplicity.

Based on the architecture of batch correlators shown in Figure 1, open-loop tracking estimates the signal code phase and Doppler frequency shift via discrete searching. Each combination of the code phase and Doppler frequency shift makes up a discrete search grid in the time-frequency two-dimension searching space. Every correlator in the architecture corresponds to one of the search grids. The correlation space, namely the code phase delay between the adjacent correlators in code dimension is the size of code search grid. The frequency difference between those in frequency dimension is the frequency search grid size. In the correlator, the input signal is correlated with the local reference signal which is composed of the carrier and code sequence generated by the Numerically Controlled Oscillators (NCO). Correlation is performed to compute the outputs of the in-phase (I) and quadrature (Q) branches as below

(2) $$I_{d,k}=\frac{1}{N_{c}}\sum_{n=0}^{N_{c}-1}s\left(t_{n}\right)c\left(t_{n}-\tau_{d}\right)\cdot \sqrt{2}cos\left(2\pi \left(f_{IF}+f_{k}\right)t_{n}\right)$$

(3) $$Q_{d,k}=\frac{1}{N_{c}}\sum_{n=0}^{N_{c}-1}s\left(t_{n}\right)c\left(t_{n}-\tau_{d}\right)\cdot \sqrt{2}sin\left(2\pi \left(f_{IF}+f_{k}\right)t_{n}\right)$$

where $N_{c}$ is the number of signal samples during the interval of coherent integration. In order to ensure signal independence, I and Q correlation values must be accumulated over an integer number of primary code periods which is 1 ms for the GPS L1CA signal and the length cannot extend the navigation data duration for the unknown transitions of navigation data when no external assisted information is introduced. However, for new GNSS signal such as GPS L5 with pilot channel, the accumulation length can be set as long as possible when the secondary code is synchronized [22]. With the help of Assisted-GPS (AGPS), the time of code phase transition induced by navigation data is known and the coherent integration can be expanded over the data duration by taking care of the transition [23]. $\tau_{d}$ and $f_{k}$ are the code phase and Doppler frequency shift of the corresponding search grid which are

(4) $$\tau_{d}=d\cdot \tau_{b}$$

(5) $$f_{k}=k\cdot f_{b}$$

where $\tau_{b}$ and $f_{b}$ are the sizes of code and frequency search grid respectively. The correlator outputs are

(6) $$I_{d,k}=\sqrt{P}R\left(\tau -\tau_{d}\right)sinc\left(\pi \left(f_{d}-f_{k}\right)T_{c}\right)cos\Delta \varphi +n_{I}$$

(7) $$Q_{d,k}=\sqrt{P}R\left(\tau -\tau_{d}\right)sinc\left(\pi \left(f_{d}-f_{k}\right)T_{c}\right)sin\Delta \varphi +n_{Q}$$

where R(·) is the autocorrelation function of the PRN code, $T_{c}$ is the coherent integration time, Δφ is the carrier phase error. $n_{I}$ and $n_{Q}$ are the noise components which are still AGWN. Their variances are uniformly expressed as below

(8) $$\begin{array}{cc} \sigma_{n,\pm }^{2} & =\frac{2}{N_{c}^{2}}\sum_{n=0}^{N_{c}-1}E\left[n_{w}^{2}\left(t_{n}\right)\cdot \frac{1\pm cos(4\pi \left(f_{IF}+f_{k}\right)t_{n})}{2}\right]\\ & =\frac{N_{0}}{2T_{c}} \end{array}$$

where E[x] is the expectation of *x*. “+” and “−” correspond to the variances of $n_{I}$ and $n_{Q}$ respectively and they are uniformly represented as $\sigma_{n}^{2}$ in the following.

The energy of the correlator outputs is computed based on I and Q values as below which makes up the entire image of the signal

(9) $$P\left(\tau_{d},f_{k}\right)=I_{d,k}^{2}+Q_{d,k}^{2}$$

The satellite signal is deemed to be in the grid with the maximum power peak in the batch correlator outputs as shown in Figure 1. The measurement of the incoming signal are calculated according to the code phase and Doppler frequency shift of the reference signal of the grid as below

(10) $$\left(\hat{\tau },\hat{f_{D}}\right)=arg\max_{\tau_{d},f_{k}}P\left(\tau_{d},f_{k}\right)$$

The estimation results $\hat{\tau }$ and $\hat{f_{D}}$ of the signal are used to form the final signal measurements. Different from the closed loop that there is a limitation in the power accumulation length owning to the requirement of the system stability, the power accumulation length of the open-loop tracking is not limited. It can be lengthened to hundreds of micro seconds and the sensitivity can be greatly improved. In this paper, the coherent integration length of 300 ms is taken as an example and the sensitivity is sufficient for the urban application [18]. For other values, although the detail parameters may change, the basic conception is not altered and the analysis results are the same.

Nevertheless, there is a serious drawback for the open loop tracking method, which is the degraded measurement accuracy. As the measurement of open loop tracking is calculated from the parameters of the discrete search grid of the detected peak, the uncertainty in the grid will probably deteriorate the measurement accuracy. If the signal is detected in the false grid, the measurement error will be even further enlarged. Compared with the Doppler frequency measurement, the problem to code phase measurement is more serious.

For Doppler frequency, considering the long coherent integration length of open loop tracking, the width of the frequency search grid is limited to an extremely small value due to the large power loss brought by the frequency mismatch [21]. If the coherent integration is as long as 300 ms as the statement above, the frequency search grid size is at most 5 Hz. Converted to velocity in the unit of m/s, taking the center frequency 1575.42 MHz of GPS L1CA signal as an example, the uncertainty within the grid is at most 0.48 m/s. Furthermore, the false detection in the frequency dimension will almost never occur because even the neighboring grids of the right one contain little signal power. Accordingly, the effect of the discrete searching on the Doppler frequency measurement accuracy is a little.

For the code phase measurement, the situation is really different. In the autocorrelation function of the PRN code, signal power is contained within the code phase delay from −1 chip to 1 chip and it has nothing to do with the coherent integration length. Considering the code chip duration in distance is as long as hundreds of meters, the errors induced by the uncertainty in the grid and false detection will both exist and considerably degrade the accuracy. Since both the two errors are related to the code search step of open loop tracking, the code search grid size should be specially chosen to improve the measurement accuracy. Additionally, in the architecture, each combination of the parameters needs a correlator to compute the signal power. Small grid size means large numbers of correlators are needed and leads to huge computational loads. Some kinds of parallel correlation computations which use joint time-frequency domain techniques such as direct and inverse discrete Fourier transforms (DFT and IDFT, accordingly) have been utilized to reduce the correlators demanded [24]. However, it is usually used for frequency searching. For code searching, it is not suitable since the extremely long power accumulation of open loop tracking will tremendously increase the scale of FFT and it is much more complex than the architecture of batch correlators. Therefore, computational loads should be considered together with the measurement accuracy improvement.

## 3. ACSA-OLTA

It is known that the positioning accuracy of GNSS is directly related to the satellite pseudo range accuracy. Since pseudo range is calculated from the code phase measurement, the code phase measuring accuracy of open loop tracking is concerned in this section. Firstly, we study the code phase measurement errors of open loop tracking with different code search grid sizes and the equivalent weight pseudo range error (EWPRE) is proposed to obtain the optimal grid size. Then, the code phase measuring error of different measurement calculation methods are assessed and compared with each other. Finally, based on the analysis, the adaptive correlation space adjusted open loop tracking Algorithm (ACSA-OLTA) is proposed to improve the pseudo range measuring accuracy. Since carrier frequency is much larger than code rate, the effect of carrier Doppler frequency error on the code phase measurement accuracy is little. Moreover, considering the carrier Doppler frequency estimation error is really small, it is ignored and assumed to be zero in the following.

### 3.1. The Optimal Correlation Space Based on EWPRE

Based on the generalized open-loop signal tracking method described earlier, the accuracy of the code phase measurement heavily relies on the code search grid size. If the grid size is too large, the uncertainty in the grid will distort the accuracy. On the contrary, if the grid size is too small, the accuracy may be greatly destroyed by the false peak detection. Since the length of the PRN code chip in distance is really large such as GPS L1CA signal whose code chip is as long as 300 m, the grid size should be much smaller than the code chip duration to guarantee the accuracy. If the peak with the most accurate measurement is called most accurate peak, the correlation outputs beside the most accurate peak contain partial signal power. Thus, the powers of these correlation outputs are easier to be larger than the most accurate peak and false peak detection will probably occur. It is obvious that the smaller the grid is, the more signal power the adjacent grids contains and the more likely the false detection occurs.

Therefore, the balance between the accuracy degradation induced by the uncertainty in the grid and false peak detection should be gained in the choice of the code search grid size. In order to obtain the optimal search grid size for the code phase measurement accuracy improvement, we propose the equivalent weight pseudo range error (EWPRE) to evaluate the measurement error and gain the balance. Both the errors brought by the uncertainty in the grid and false peak detection are involved so as to synthesize the effects of the two factors on the measurement accuracy.

In the design of EWPRE, we firstly evaluate the code phase measurement errors when the most accurate peak or false peak is detected. For the measurements that are directly extracted from the parameters of the search grid, as it is a biased estimation with the jitter limited within the grid, the mean error is utilized for the error assessment. When the most accurate peak in the signal image is detected, the code phase measurement error is derived from the uncertainty of the real code phase in the search grid. In this case, the real signal code phase obeys the uniform distribution in the search grid expressed as below [25]

(11) $$f\left(c_{e}\right)=1/\tau_{b},-\tau_{b}/2\leq c_{e}\leq \tau_{b}/2$$

Thus, the mean measurement error is

(12) $$c_{me}\left(\tau_{b}\right)=\int_{-\tau_{b}/2}^{\tau_{b}/2}\left|c_{e}\right|\cdot f\left(c_{e}\right)dc_{e}=\tau_{b}/4$$

According to the equation, it is clear that, when the most accurate peak is detected, the measurement error is proportional to the grid size and wide search grid will induce large code phase measuring error.

When the false peak is detected, besides the error induced by the uncertainty in the search grid, the deviation of the search grid against that with the most accurate measurement is involved in the measuring error. If the nth grid relative to the grid with the most accurate measurement is falsely detected, the mean measurement error is

(13) $$e_{n}\left(\tau_{b}\right)=\int_{n\tau_{b}-\tau_{b}/2}^{n\tau_{b}+\tau_{b}/2}\left|x\right|\cdot \frac{1}{\tau_{b}}dx=n\tau_{b}$$

It is obvious that the measurement error brought by false peak detection is much larger than that in the grid. Hereafter, we will develop the weight coefficients of the measurement errors when the most accurate peak or false peak is detected in the EWPRE. In order to synthetically evaluate the effects of the two kinds of errors on the measurement, the weight coefficients should be the probability that the errors occur. Usually, in open loop tracking, the peak with the largest power is regarded as the most accurate peak and the measurement will be extracted from the grid of that peak. Although prior information has been used to judge whether the peak is the most accurate one, it cannot distinguish the false peak whose corresponding code search grid is close to the most accurate peak since measurements extracted from these grids are all probable. Only power can be utilized and false detection will occur when the correlated power of the adjacent grids is larger than the most accurate peak. Therefore, the probability that the error occurs is that the false peak has power larger than the most accurate peak.

Based on the detection variable expressed in Equation (9), the correlation outputs of two grids in code dimension with the same Doppler frequency are considered. One of them is the most accurate peak, the other one is the false peak. There are two situations for the probability calculation. When the space between the girds of the two peaks in code dimension is larger than one code chip, the correlated powers of the most accurate peak and the false peak are independent from each other and only the most accurate peak contains signal power. If $P_{r}$ and $P_{f}$ are the correlated power of the most accurate peak and the false peak, according to Equation (9), $P_{r}$ obeys non-central $\chi^{2}$ (chi-square) distribution with two degrees of freedom and $P_{f}$ obeys central $\chi^{2}$ distribution with two degrees of freedom. The probability density functions (PDF) of them are

(14) $$f_{p}\left(x\right)=\frac{1}{2\sigma_{n}^{2}}exp\left(-\frac{x+\alpha^{2}}{2\sigma_{n}^{2}}\right)I_{0}\left(\frac{\alpha }{\sigma_{n}^{2}}\sqrt{x}\right)$$

(15) $$f_{a}\left(x\right)=\frac{1}{2\sigma_{n}^{2}}exp\left(-\frac{x}{2\sigma_{n}^{2}}\right)$$

where α is the non-central parameter and it is equal to $\sqrt{P}$ which is the energy of the most accurate peak. $I_{0}\left(\cdot \right)$ is the zero-order modified Bessel function of the first kind [26]. The joint PDF is

(16) $$f_{u}\left(P_{r},P_{f}\right)=f_{p}\left(P_{r}\right)\cdot f_{a}\left(P_{f}\right)$$

The probability false detection occurs is that when $P_{f}>P_{r}$ and it is expressed as

(17) $$\begin{array}{cc} & F\left(P_{f}>P_{r}\right)=\underset{P_{f}>P_{r}}{\;\int \int \;}f_{u}\left(\gamma ,\beta \right)d\gamma d\beta \\ = & exp\left(-\frac{\alpha^{2}}{4\sigma_{n}^{2}}\right)\cdot \int_{0}^{+\infty }\frac{1}{2\sigma_{n}^{2}}exp\left(-\frac{2\beta +\alpha^{2}/2}{2\sigma_{n}^{2}}\right)I_{0}\left(\frac{\alpha /\sqrt{2}}{\sigma_{n}^{2}}\sqrt{2\beta }\right)d\beta \\ = & \frac{1}{2}exp\left(-\frac{\alpha^{2}}{4\sigma_{n}^{2}}\right) \end{array}$$

When the space between the girds of the most accurate peak and the false peak in code dimension is smaller than one code chip, $P_{r}$ and $P_{f}$ are not independent from each other and both of them contain signal power. Let $I_{r}$ and $Q_{r}$ be the in-phase and quadrature branch correlation outputs of the most accurate peak and $I_{f}$ and $Q_{f}$ be the in-phase and quadrature branch correlation outputs of the false peak, supposing the Doppler frequency error is zero, their expressions are as below according to Equations (6) and (7)

(18) $$I_{r}=\sqrt{P_{r}}cos\Delta \varphi_{1}=\sqrt{P}R\left(\tau -\tau_{d}\right)cos\Delta \varphi_{1}+n_{I,r}$$

(19) $$Q_{r}=\sqrt{P_{r}}sin\Delta \varphi_{1}=\sqrt{P}R\left(\tau -\tau_{d}\right)sin\Delta \varphi_{1}+n_{Q,r}$$

(20) $$I_{f}=\sqrt{P_{f}}cos\Delta \varphi_{2}=\sqrt{P}R\left(\tau -\tau_{d}-\Delta \tau_{b}\right)cos\Delta \varphi_{2}+n_{I,f}$$

(21) $$Q_{f}=\sqrt{P_{f}}sin\Delta \varphi_{2}=\sqrt{P}R\left(\tau -\tau_{d}-\Delta \tau_{b}\right)sin\Delta \varphi_{2}+n_{Q,f}$$

where $\Delta \tau_{b}$ is the code phase space between the two peaks and $|\Delta \tau_{b}|<1\;code\;chip$. $\Delta \varphi_{1}$ and $\Delta \varphi_{2}$ are the carrier phase residuals. $n_{I,r}$, $n_{Q,r}$, $n_{I,f}$ and $n_{Q,f}$ are the noise components. They are AWGN and the variances of them are all $\sigma_{n}^{2}$ according to Equation (8). Because of the orthogonal carrier phase relationship, $n_{I,r}$ and $n_{I,f}$ are independent of $n_{Q,r}$ and $n_{Q,f}$. The covariance between $n_{I,r}$ and $n_{I,f}$ is

(22) $$\begin{array}{cc} cov(n_{I,r},n_{I,f};\Delta \tau_{b}) & =\frac{1}{N_{c}^{2}}E\left[n_{w}^{2}\left(t_{n}\right)\left(1+cos\left(4\pi \left(f_{IF}+f_{d}\right)t_{n}\right)\right)\right]E\left[\sum_{n=0}^{N_{c}-1}c\left(t_{n}-\tau_{d}\right)c\left(t_{n}-\tau_{d}-\Delta \tau_{b}\right)\right]\\ & =\sigma_{n}^{2}\cdot R\left(\Delta \tau_{b}\right)\;\left(\Delta \tau_{b}<1\;code\;chip\right) \end{array}$$

The covariance between $n_{Q,r}$ and $n_{Q,f}$ is

(23) $$\begin{array}{cc} cov(n_{Q,r},n_{Q,f};\Delta \tau_{b}) & =\frac{1}{N_{c}^{2}}E\left[n_{w}^{2}\left(t_{n}\right)\left(1-cos\left(4\pi \left(f_{IF}+f_{d}\right)t_{n}\right)\right)\right]E\left[\sum_{n=0}^{N_{c}-1}c\left(t_{n}-\tau_{d}\right)c\left(t_{n}-\tau_{d}-\Delta \tau_{b}\right)\right]\\ & =\sigma_{n}^{2}\cdot R\left(\Delta \tau_{b}\right)\;\left(\Delta \tau_{b}<1\;code\;chip\right) \end{array}$$

Thus, $I_{r}$, $I_{f}$, $Q_{r}$ and $Q_{f}$ are all Gaussian random variables. According to the definition of the variables $\left[I_{r},I_{f},Q_{r},Q_{f}\right]$, the covariance matrix K is given by

(24) $$K=\left[\begin{array}{cccc} \sigma_{n}^{2} & \sigma_{n}^{2}\cdot R\left(\Delta \tau_{b}\right) & 0 & 0\\ \sigma_{n}^{2}\cdot R\left(\Delta \tau_{b}\right) & \sigma_{n}^{2} & 0 & 0\\ 0 & 0 & \sigma_{n}^{2} & \sigma_{n}^{2}\cdot R\left(\Delta \tau_{b}\right)\\ 0 & 0 & \sigma_{n}^{2}\cdot R\left(\Delta \tau_{b}\right) & \sigma_{n}^{2} \end{array}\right]$$

Supposing $P_{r,f}={\left[I_{r},I_{f},Q_{r},Q_{f}\right]}^{T}$, the joint PDF is

(25) $$f\left(I_{r},I_{f},Q_{r},Q_{f};\Delta \tau_{b}\right)=\frac{1}{{\left(2\pi \right)}^{2}\sqrt{\left|K\right|}}exp\left(-\frac{1}{2}\left(P_{r,f}-\mu \right)K^{-1}{\left(P_{r,f}-\mu \right)}^{T}\right)$$

where $\mu ={\left[\mu_{I_{r}},\mu_{I_{f}},\mu_{Q_{r}},\mu_{Q_{f}}\right]}^{T}$ and it is the mean value vector of $P_{r,f}$. By computing the inverse matrix of K, the expression of the joint PDF is

(26) $$\begin{array}{cc} f(I_{r},I_{f}, & Q_{r},Q_{f};\Delta \tau_{b})=\frac{1}{{\left(2\pi \right)}^{2}\cdot \sigma_{n}^{4}\left(1-R^{2}\left(\Delta \tau_{b}\right)\right)}exp(-\frac{1}{2\sigma_{n}^{2}\left(1-R^{2}\left(\Delta \tau_{b}\right)\right)}\\ & [{\left(I_{r}-\mu_{r,I}\right)}^{2}+{\left(I_{f}-\mu_{f,I}\right)}^{2}-2R\left(\Delta \tau_{b}\right)\left(I_{r}-\mu_{r,I}\right)\left(I_{f}-\mu_{f,I}\right)\\ & +{\left(Q_{r}-\mu_{r,Q}\right)}^{2}+{\left(Q_{f}-\mu_{f,Q}\right)}^{2}-2R\left(\Delta \tau_{b}\right)\left(Q_{r}-up\mu_{r,Q}\right)\left(Q_{f}-\mu_{f,Q}\right]) \end{array}$$

Submitting $I_{r}=\sqrt{P_{r}}cos\left(\Delta \varphi_{1}\right)$, $Q_{r}=\sqrt{P_{r}}sin\left(\Delta \varphi_{1}\right)$, $I_{f}=\sqrt{P_{f}}cos\left(\Delta \varphi_{2}\right)$ and $Q_{f}=\;\sqrt{P_{f}}sin\left(\Delta \varphi_{2}\right)$ into Equation (26), after some algebra manipulations, the expression of the joint PDF of $P_{r}$ and $P_{f}$ is

(27) $$\begin{array}{cc} f & \left(P_{r},P_{f};\Delta \tau_{b}\right)=\frac{1}{{\left(2\pi \right)}^{2}\cdot \sigma_{n}^{4}\left(1-R^{2}\left(\Delta \tau_{b}\right)\right)}exp(-\frac{1}{2\sigma_{n}^{2}}(P_{r}+P_{f}+\mu_{I_{r}}^{2}+\mu_{I_{f}}^{2}+\mu_{Q_{r}}^{2}+\mu_{Q_{f}}^{2}\\ & -2R\left(\Delta \tau_{b}\right)\left(\mu_{I_{r}}\mu_{I_{f}}+\mu_{Q_{r}}\mu_{Q_{f}}\right)))\times \int_{0}^{2\pi }\int_{0}^{2\pi }exp(\frac{\sqrt{P_{r}\cdot P_{f}}}{\sigma_{n}^{2}}R\left(\Delta \tau_{b}\right)cos\left(\Delta \varphi_{1}-\Delta \varphi_{2}\right)\\ & +\frac{\sqrt{P_{r}}}{\sigma_{n}^{2}}\left(\mu_{f,I}R\left(\Delta \tau_{b}\right)-\mu_{r,I}\right)cos\left(\Delta \varphi_{1}\right)+\frac{\sqrt{P_{f}}}{\sigma_{n}^{2}}\left(\mu_{r,I}R\left(\Delta \tau_{b}\right)-\mu_{f,I}\right)cos\left(\Delta \varphi_{2}\right)\\ & +\frac{\sqrt{P_{r}}}{\sigma_{n}^{2}}\left(\mu_{f,Q}R\left(\Delta \tau_{b}\right)-\mu_{r,Q}\right)sin\left(\Delta \varphi_{1}\right)+\frac{\sqrt{P_{f}}}{\sigma_{n}^{2}}\left(\mu_{r,Q}R\left(\Delta \tau_{b}\right)-\mu_{f,Q}\right)sin\left(\Delta \varphi_{2}\right))d\Delta \varphi_{1}d\Delta \varphi_{2} \end{array}$$

When $\Delta \tau_{b}$ varies from 0 to 1 chip, the probability that false detection occurs is

(28) $$F\left(P_{f}>P_{r};\Delta \tau_{b}\right)=\int_{0}^{+\infty }\left[\int_{0}^{P_{f}}f\left(P_{r},P_{f}\right)dP_{r}\right]dP_{f}$$

**Figure 2:**
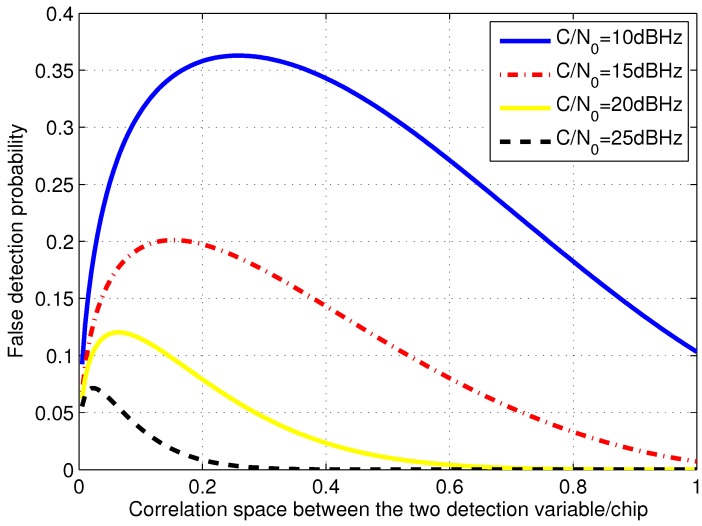
False detection probability of different correlation space and $C/N_{0}$.

Taking the coherent integration length of 300 ms as an example, the false detection probability for different $\Delta \tau_{b}$ and different carrier-to-noise density ratio ($C/N_{0}$) is shown in Figure 2. It is obvious the false peak detection probability is related to the space between the most accurate peak and the false peak. Since more signal power is contained in the false peak when the space is small, the probability increases as the space between the two peaks decreases. While, when the space is too small, the probability become lower because of the significant positive correlation between the powers of the two peaks according to Equations (22) and (23). Hence, when the grid size is small, the false peak detection will probably occur especially at the point with the largest false detection probability. However, large grid size contains large inherent measurement error. In addition, when the correlation space exceeds 1 code chip, the false peak detection probability is extremely small with respect to those within the code chip duration so that it is not considered in the following analysis. In consideration that open-loop tracking extracts code phase measurement from that of the grid where the detected peak is, the results further validate that the search grids with the size too large or two small will both degrades the measurement accuracy.

Furthermore, it also shows that the false detection probability becomes high as ($C/N_{0}$) decreases. When $C/N_{0}$ is high, the most accurate peak with the maximum signal power is easily detected from the false peak. While, when $C/N_{0}$ is low, both the two peaks are greatly disturbed by noise so that they are difficult to be distinguished and false detection will probably occur. In addition, the code phase spaces with the largest false detection probability are various among different $C/N_{0}$ due to the positive correlation between the two peaks besides the signal power. The lower the $C/N_{0}$ is, the larger the corresponding space with the highest false detection probability is. Therefore, the signal power should be considered in the choice of code search grid size.

Considering that the false peak detection probabilities can indicate the possibility of the occurrence of the measurement errors induced by the false detection at different points, the weight coefficients are designed based on the probability. The weight coefficient for the nth grid relative to grid with the most accurate measurement is expressed as below

(29) $$W_{n}\left(\tau_{b}\right)=\left\{\begin{array}{ccc} & \frac{1}{T_{p}}\int_{0}^{\frac{\tau_{b}}{2}}dx+\frac{1}{T_{p}}\int_{\frac{\tau_{b}}{2}}^{T_{p}}\left(1-F\left(P_{f}>P_{r};x\right)\right)dx & n=0\\ & \frac{1}{T_{p}}\int_{\frac{2n-1}{2}\tau_{b}}^{\frac{2n+1}{2}\tau_{b}}F\left(P_{f}>P_{r};x\right)dx & n=1,2,... \end{array}\right.$$

where n=0 corresponds to the grid with the most accurate measurement. $T_{p}$ is the code chip duration. The weighed coefficients are the integration of the probability that the detection result is in the false grid or in the right grid. Accordingly, it evaluates the likelihood of the measurement errors corresponding to the detection of the correct or false peak. Based on the weight coefficients, the EWPRE is

(30) $$\begin{array}{c} S\left(\tau_{b}\right)=W_{0}\left(\tau_{b}\right)\cdot c_{me}\left(\tau_{b}\right)+\sum_{n=1}^{T_{p}/\tau_{b}}\left[W_{n}\left(\tau_{b}\right)\cdot e_{n}\left(\tau_{b}\right)\right] \end{array}$$

It is the weighed sum of the code phase measurement errors when the detection results vary from the grid of the most accurate peak to that one code chip away. The measurement error induced by the false detection in other grids and that when the most accurate peak is detected are both involved and weighed to accumulate together. With the help of the weight coefficients, when the false detection probability is large, the measurement error induced by the false peak detection will be dominant in the weighted error. On the contrary, the measurement error within the grid will be more significant. As a result, it synthesizes the effects of the uncertainty in the search grid and false peak detection on the code phase measurement accuracy. The value of EWPRE represents the mean equivalent relative measurement error among different grid sizes so that it can evaluate the measurement accuracy difference of these grid sizes even though it is not the estimation of the real mean measurement error. It is further verified by simulation in Section 4.1.

**Figure 3:**
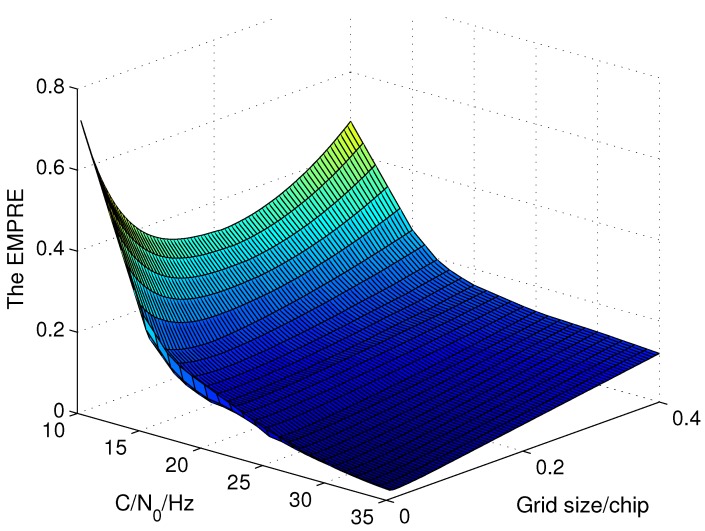
The EWPRE of different grid sizes and $C/N_{0}$ with 300 ms coherent integration.

Consequently, the optimal code search grid size of open loop tracking method for a given $C/N_{0}$ is the one with the minimum EWPRE. After some algebraic transformation, the EWPRE is simplified as below. When the coherent integration length is 300 ms, the EWPRE of different grid sizes for different $C/N_{0}$ is shown in Figure 3.

(31) $$S\left(\tau_{b}\right)=\frac{\tau_{b}^{2}}{8T_{p}}+\frac{1}{T_{p}}\sum_{n=1}^{T_{p}/\tau_{b}}\left[\left(e_{n}-\tau_{b}/4\right)\cdot \int_{\frac{2n-1}{2}\tau_{b}}^{\frac{2n+1}{2}\tau_{b}}F\left(P_{f}>P_{r};x\right)dx\right]$$

**Figure 4:**
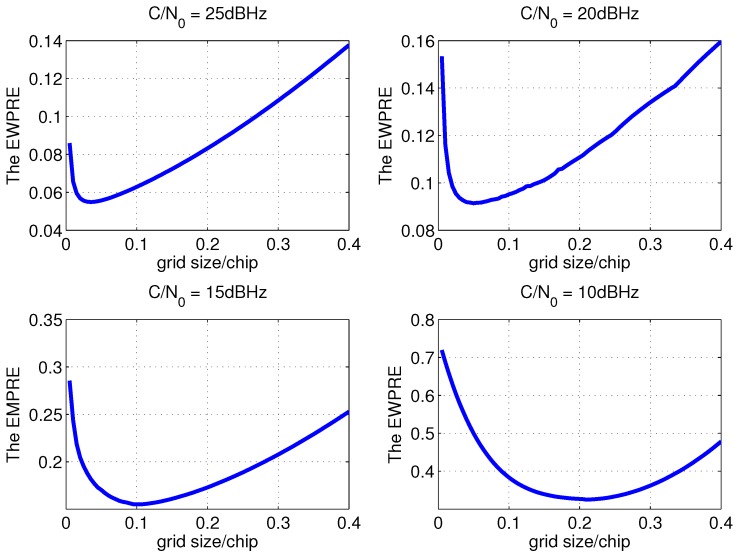
The detailed EWPRE of different grid size with 300 ms coherent integration.

Taking the $C/N_{0}$ of 10 dBHz, 15 dBHz, 20 dBHz and 25 dBHz as examples, the EWPRE of different grid sizes is detailedly drawn in Figure 4.

**Figure 5:**
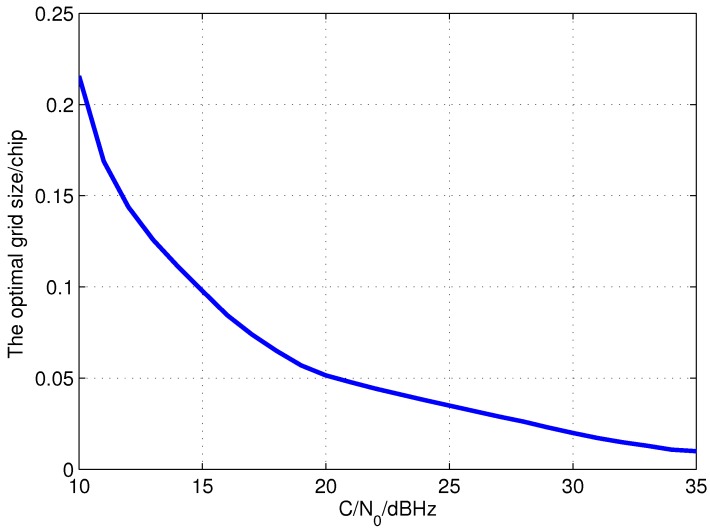
Optimal code search grid size of different $C/N_{0}$ with 300 ms coherent integration.

One can see that there is a grid size that minimizes the EWPRE for each $C/N_{0}$ and that shall be the grid size with best accuracy. Taking the coherent integration length of 300 ms as an example, the optimal code search grid sizes of different $C/N_{0}$ are shown in Figure 5. It indicates that when $C/N_{0}$ is low, the grid size is enlarged to increase the power difference between adjacent grids so as to avoid the error brought by the false peak detection which is much more serious than the error within the grid. When the $C/N_{0}$ is high, as the detection performance is perfect, the optimal grid size is really small to reduce the measurement error in the grid. Therefore, the correlation space between the batch correlators of open loop tracking which is the code search grid size should be adjusted to the optimal size shown in Figure 5 as $C/N_{0}$ varies to improve the measurement accuracy. Considering small grid size demands large numbers of correlators which means huge computational costs, the method to calculate the measurement with less correlators but high accuracy is studied in the next subsection. The verification of the optimal grid size is conducted in Section 4 with the help of Monte Carlo simulation and test.

### 3.2. The Measurement Calculation Strategy

In order to reduce the correlators needed and keep the accuracy at the same time, early-minus-late discriminator (EMLD) is introduced [8]. However, no previous study has analyzed the accuracy of the method. In this section, the accuracy of the measurement derived from the discriminator is evaluated. Based on the accuracy comparison between the code phase calculated with the EMLD method and that directly extracted from the local reference signal of the detected grid, together with the computational cost assessment results, the measurement calculation strategy is developed.

The EMLD based code phase estimation begins after the peak detection. The correlation outputs of the two code search grids adjoining the peak are used to construct the discriminator. If the two grids are represented by *E* and *L* corresponding to that with the code phase earlier or later than the peak respectively, the in-phase and quadrature correlation outputs of the two grids are expressed as below

(32) $$\begin{array}{cc} & E_{I}=\sqrt{P}R\left(\tau -k\tau_{b}-\tau_{b}\right)cos\left(\Delta \varphi \right)+n_{I,E}\\ & E_{Q}=\sqrt{P}R\left(\tau -k\tau_{b}-\tau_{b}\right)sin\left(\Delta \varphi \right)+n_{Q,E}\\ & L_{I}=\sqrt{P}R\left(\tau -k\tau_{b}+\tau_{b}\right)cos\left(\Delta \varphi \right)+n_{I,L}\\ & L_{Q}=\sqrt{P}R\left(\tau -k\tau_{b}+\tau_{b}\right)sin\left(\Delta \varphi \right)+n_{Q,L} \end{array}$$

where $n_{I,E}$, $n_{Q,E}$, $n_{I,L}$ and $n_{Q,L}$ are the noise components in the correlation results. *k* is the grid index of the detected peak. The final code phase estimation result $\tau_{f}$ is

(33) $$\tau_{f}=k\tau_{b}+\frac{1}{1-2\tau_{b}}\frac{\left(E_{I}^{2}+E_{Q}^{2}\right)-\left(L_{I}^{2}+L_{Q}^{2}\right)}{\left(E_{I}^{2}+E_{Q}^{2}\right)+\left(L_{I}^{2}+L_{Q}^{2}\right)}$$

In this way, regardless of the noise, the discriminator can completely compensate the uncertainty of the code phase in the detected grid [8]. If the most accurate peak is detected, the code phase estimation is totally unbiased. However, noise will distort the code phase measurement so that the error standard deviation is utilized for the evaluation. Assuming that it is perfectly normalized discriminator and the front-end bandwidth is infinite, the variance of the measurement calculated from the discriminator is as below (details of derivation are shown in the appendix)

(34) $$\sigma_{EML}^{2}=\frac{\tau_{b}}{2\cdot C/N_{0}\cdot T_{c}}\left(1+\frac{1}{C/N_{0}\cdot T_{c}\left(1-\tau_{b}\right)}\right)$$

At the same time, for the code phase that directly extracted from the grid parameters, when the most accurate peak is detected, the variance of the measurement is

(35) $$\sigma_{c}^{2}=\int_{-\tau_{d}/2}^{\tau_{d}/2}c_{e}^{2}\cdot f\left(c_{e}\right)dc_{e}=\tau_{b}^{2}/12$$

In the following, we use the abbreviations below to represent the open loop tracking with the two different code phase measurement calculation methods.

- DIS: The open-loop tracking with EMLD dependent code phase estimation method
- DET: The open-loop tracking that directly calculate the code phase from the grid parameters.

**Figure 6:**
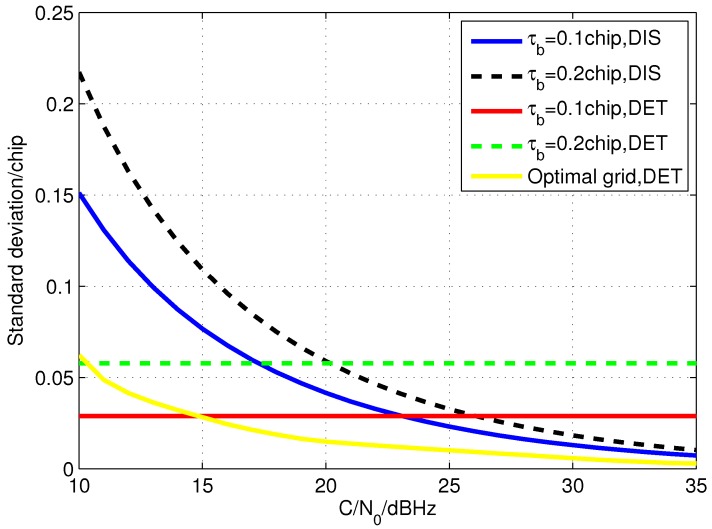
The code phase error standard deviation of the two measurement calculation methods without consideration of false peak detection.

Assuming that the peak detection is correct, the error standard deviations of measurements calculated by DIS and DET with different grid sizes are drawn in Figure 6 in which the coherent integration length is 300 ms. It shows that, when $C/N_{0}$ is high, the accuracy of DIS is much better than that of DET with the same grid size. Furthermore, although the accuracy of DET with optimal grid size is better than the discriminator based method with the grid size of 0.1 chip when $C/N_{0}$ is high, the improvement is a little and it needs a huge number of correlators. However, as the $C/N_{0}$ decreases, the accuracy of DIS is destroyed and it becomes worse than DET. Moreover, if false peak detection occurs, the accuracy of DIS will be further distorted. Thus, the EMLD method cannot be utilized all the time as that in [19] and a $C/N_{0}$ threshold is needed to switch the code phase calculation methods.

Taking the coherent integration length of 300 ms as an example, if the $\tau_{b}$ of DIS is 0.1 chip and DET has the optimal grid size shown in Figure 5, when $C/N_{0}$ is larger than 23 dBHz which is the standard deviation cross point of the measurements derived from the two methods, the correlators needed to search the area of 1 chip and the corresponding measurement error standard deviation are listed in Table 1.

**Table 1:** The statistic of the correlator needed and the mean accuracy.

$C/N_{0}$ (dBHz)		23	24	25	26	27	28	29	30
DET	correlator number	24.3	26.2	28.5	30.9	34.7	38.6	44.0	50.3
	standard deviation (chip)	0.0119	0.0110	0.0101	0.0094	0.0083	0.0075	0.0066	0.0057
DIS	correlator number	10	10	10	10	10	10	10	10
	standard deviation (chip)	0.0292	0.0260	0.0231	0.0206	0.0183	0.0163	0.0145	0.0129

In the table, the measurement accuracy is calculated under the assumption that the peak detection is correct. Since the optimal grid size is smaller than 0.1 chip when $C/N_{0}$ is larger than 23 dBHz, the false peak detection probability of the open loop with the optimal grid size is a little larger than that with the grid size of 0.1 chip so that the code phase measuring error is more coincident. As a result, the measurement accuracy of DET and DIS in these cases are more or less the same which is further verified in Section 4.1. In consideration that DIS can hugely decrease the correlators needed, it is preferred when $C/N_{0}$ is high. When $C/N_{0}$ reduces, the measurement accuracy of DIS is quickly decreased due to the noise and it is much poorer than the DET with the optimal grid size. For the more, as $C/N_{0}$ decreases, with the grid size of 0.1 chip, the false detection probability become large and the accuracy will be further distorted. In fact, when $C/N_{0}$ is lower than 23 dBHz, the measurement accuracy of DIS will quickly degrade which is validated by the simulation results in Section 4.1. Therefore, we propose the measurement calculation strategy that switches the two code phase calculation methods at the $C/N_{0}$ of 23 dBHz, namely the standard deviation cross point of the measurements computed with the two methods. In the strategy, when $C/N_{0}$ is higher than 23 dBHz, the discriminator is used and the grid size is 0.1 chip. When $C/N_{0}$ is lower than 23 dBHz, measurement is calculated directly based on the parameters of grid with the optimal size.

### 3.3. Adaptive Correlation Space Adjusted Method

In view of the analysis above, the optimal code search grid size is different when the received signal power varies. For the purposes to improve the code phase measurement accuracy of open loop tracking in urban area, the adaptive correlation space adjusted open loop tracking approach (ACSA-OLTA) is proposed. In ACSA-OLTA, the correlation space between the batch correlators is adjusted to the optimal code search grid size calculated from the EWPRE according to $C/N_{0}$ which displays the signal power [20]. The EMLD based measurement calculation method is utilized at the proper $C/N_{0}$ to further improve the accuracy and reduce the number of correlator needed at the same time. In addition, the search grid size in frequency dimension is set to a fixed value. When the grid size in code dimension varies according to the $C/N_{0}$, the size in frequency dimension is unchanged. The Doppler frequency measuring result of the satellite signal is that of the local reference signal of the detected grid.

Taking the coherent integration of 300 ms as an example, the search grid size in frequency dimension is 5 Hz. The code search grid sizes for different $C/N_{0}$ are set as those shown in Figure 5. The process of the method is summarized as below

Step 1: Initialize the code search grid size as 0.2 chip, Doppler frequency grid size as 5 Hz. Then, carry out the coherent integration in the batch correlators.
Step 2: After the coherent integration, detect the signal in the image of the correlator outputs. Estimate the $C/N_{0}$ of the incoming signal with the help of the correlation result of the detected peak.
Step 3: Adjust the correlation space between adjacent correlators according to the estimated $C/N_{0}$. When $C/N_{0}$ is larger than 23 dBHz, it is set to 0.1 chip. Or else, it is set as Figure 5. The signal code phase and Doppler frequency are directly extracted from the grid and fed back to the local signal generators.
Step 4: Correlate the incoming signal with the new local replicas to form the signal image.
Step 5: After removing the multipath peaks and other improper peaks according to the prior information, the peak with the maximum power in the image is selected.
Step 6: The code phase is calculated by EMLD when $C/N_{0}$ is higher than 23 dBHz. Otherwise, the code phase is that of the grid. The estimated parameters are fed back to the local signal generators.
Step 7: Estimate the $C/N_{0}$ with the correlation output of the detected peak. Then, adjust the correlation space as Figure 5 if $C/N_{0}$ is lower than 23 dBHz, or else it is set to 0.1 chip. Return to Step 4.

For other proper coherent integration lengths, the process is probably the same. The only differences are the switch point of the measurement calculation methods and the optimal grid sizes. Since all of these parameters can be calculated based on the methods above, they are not detailed for simplicity.

In the method, at the beginning, the signal power is unknown and the correlation space has to be set to an empirical value. In order to keep the correctness of peak detection, the code search grid size is initialized as 0.2 chip which is relatively large. In this way, the most accurate peak can be detected easily in which most signal power is contained. The $C/N_{0}$ will be perfectly estimated with the corresponding correlation outputs. As the code phase of the detected peak is always aligned with the incoming signal when the correlation space is varying, almost all signal power will be involved in the peak all the time and adjusting the space has tiny effects on $C/N_{0}$ estimation. The details of $C/N_{0}$ estimation have been given in [21,27] which can provide accurate $C/N_{0}$ estimation so that it is not described here for simplicity.

With the help of the $C/N_{0}$ estimation, the correlation space of the batch correlators in open loop tracking is adjusted to the optimal one calculated from EWPRE. The measurement calculation methods are also changed according to the $C/N_{0}$. In this way, as the satellite signal power is continuously varying in the real application area due to the sheltering, scattering, etc, the proposed ACSA-OLTA can greatly improve the code phase measurement accuracy of open loop tracking. The performance of ACSA-OLTA is evaluated by tests in the next section.

## 4. Performance Evaluation

In this section, we firstly evaluate the code phase measurement accuracy of open-loop tracking method with the code search grid sizes shown in Figure 5 for different $C/N_{0}$ and verify the grid size calculated based on EWPRE is optimal. Afterwards, the performance of the proposed ASCA-OLTA is assessed with the GPS L1CA signal data collected in open air area by off-line processing. Since positioning accuracy is directly related to the pseudo range calculated from the code phase measurement, by utilizing the data collected in the open air area, the positioning accuracy of the open loop tracking methods with different inherent detection grid sizes are compared with the proposed method when $C/N_{0}$ varies. The method that uses EMLD is also considered here. Finally, with the help of the data collected from the urban area, the total performance of these methods in urban area is evaluated.

### 4.1. Accuracy of the Code Phase Measurement

In order to verify that the grid sizes gained from EWPRE bring the optimal code phase measuring accuracy for the corresponding $C/N_{0}$ in the presence of AGWN, Monte Carlo simulation is carried out to evaluate the measurement accuracy of open loop tracking method. In the simulation, the measurement is directly extracted from the grid. The mean measurement error of the open loop tracking is computed to testify that the EWPRE can exactly indicate the difference of the measuring accuracy among different grid sizes. The expression of the mean measurement error is

(36) $$E_{m}=\frac{1}{N_{t}}\sum_{n=1}^{N_{t}}\left|\tau_{t,n}-\hat{\tau_{t,n}}\right|$$

where $N_{t}$ is the total number of the tests which is 5000 in the simulation. $\tau_{t,n}$ is the real code phase of the nth test and $\hat{\tau_{t,n}}$ is that derived from the open loop tracking. Then, the code phase measurement error standard deviations of open loop tracking with the optimal grid size and other grid sizes are calculated for different $C/N_{0}$. The simulation parameters are shown in Table 2. The front-end filtering, quantization and other non-idealities are not considered for analytical simplicity. The Doppler frequency error is assumed to be zero since it almost has nothing to do with the code phase measurement accuracy.

**Figure 7:**
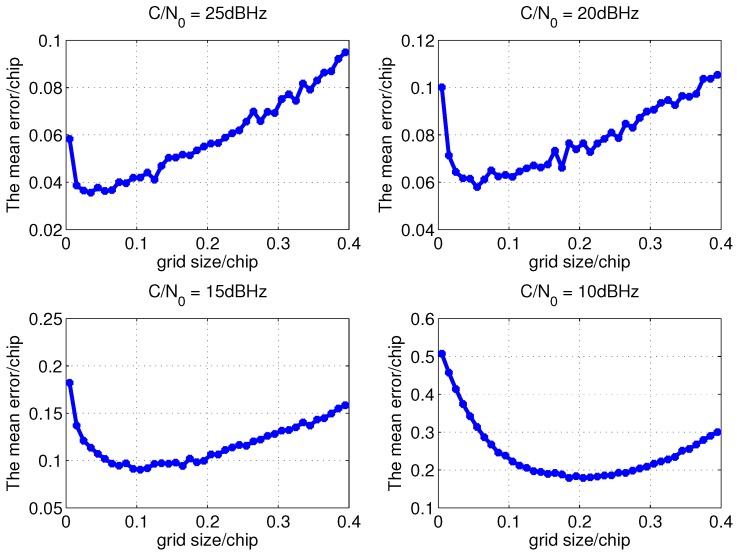
The mean code phase measurement error.

Taking the $C/N_{0}$ of 10 dBHz, 15 dBHz, 20 dBHz and 25 dBHz as examples, the simulation results of the mean code phase measurement error of open loop tracking method with the code search grid sizes listed in Table 2 is shown in Figure 7. It is clear that the shape of the mean error curves in the figure are more or less the same as those in Figure 4 and the grid sizes with the minimum mean measurement error are the same as those with minimum EWPRE. For other $C/N_{0}$, the simulation results demonstrate that the grid size calculated from EWPRE brings the best accuracy as well. As a result, it verified that the grid size derived from EWPRE is optimal.

**Table 2:** The parameters of the code phase accuracy simulation.

Parameters	Value
Signal	GPS L5 pilot signal
Signal $C/N_{0}$	10 dBHz~35 dBHz
Signal $C/N_{0}$ step	1 dB
Signal sampling rate	62 MHz
Code search grid size	0.01 chip~0.4 chip
Code search grid size step	0.01 chip
Coherent integration length	300 ms
Doppler frequency error	0 Hz
Code phase	Uniformly distributed in the range of 2 adjacent chip

**Figure 8:**
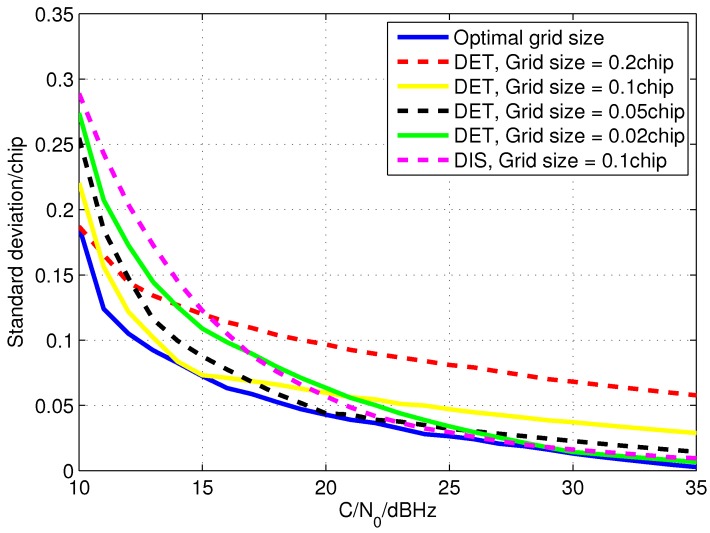
The standard deviation of the code phase measuring error for different $C/N_{0}$.

Figure 8 illustrates the simulation result of the code phase measurement error standard deviation for the open loop tracking method with the optimal code search grid size that is calculated in Section 3.1. The standard deviations of the code phase measurement error of open loop tracking with the grid sizes of 0.2 chip, 0.1 chip, 0.05 chip and 0.02 chip are drawn for comparison. It confirmed that the optimal code search grid sizes show higher measurement accuracy than the other grid sizes in the condition of the same $C/N_{0}$. Therefore, by changing the grid size to the optimal ones according to $C/N_{0}$, the accuracy of the code phase measurement can be greatly improved. In addition, the EMLD based open loop tracking method with the grid size of 0.1 chip is simulated as well. The error standard deviation of the code phase measurements computed by the EMLD is also drawn in the figure. It demonstrates that the accuracy of the method is more or less the same as that directly extracted from the grid with optimal size when $C/N_{0}$ is high. However, it will be quickly degraded when $C/N_{0}$ is lower than 20 dBHz which is much poorer than the measurement directly extracted from the grid.

### 4.2. Positioning Accuracy Test

**Figure 9:**
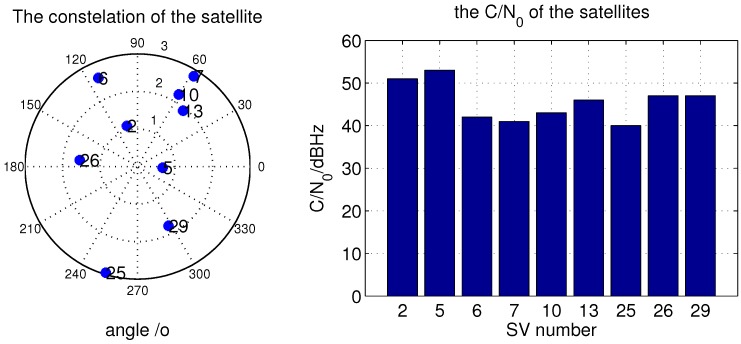
Constellation and $C/N_{0}$ of the satellites.

In this section, the ACSA-OLTA based positioning accuracy is evaluated by the off-line processing with the help of the GPS satellite signals. Since few satellites send new GNSS signals, the GPS L1CA signal is used in the test. In order to clearly confirm the experiment condition, also to correctly obtain the navigation message in the signal to eliminate the phase inversion and lengthen the coherent integration length as the pilot signal, all of the origin satellite signals with a relatively high $C/N_{0}$ are collected in the open air in the Beijing Institute of Technology campus. The constellation and $C/N_{0}$ are showed in Figure 9. The $C/N_{0}$ is gained from a NovAtel navigation receiver. The Geometric Dilution of Precision (GDOP), which is a geometry factor, representing the amplification of the standard deviation of the measurement errors onto the solution [21], is less than 2.0 during the collection.

In the first place of the test, the navigation message is collected by processing the original satellite data. The evaluation is carried out after the message in the GPS L1CA signal is demodulated based on the collected one. The coherent integration length of open-loop tracking in the test is 300 ms. The frequency search step is 5 Hz. For other feasible values, the comparison results are more or less the same so that they are not listed for simplicity. In this case, as the tests of the open loop tracking methods with different measurement extraction strategies are based on the same data, at any time during the test, the satellites that can be detected and used for positioning are all the same among these methods. Hence, the satellite constellation, ephemeris error, atmosphere error and $C/N_{0}$ are all identical in the test. The pseudo range based Least square (LS) positioning algorithm is used to test the accuracy for the reason that there is no filter in the process of LS and it can better reveal the variation of the measurement [21]. Accordingly, the positioning accuracy can remarkably indicate the code phase measuring performance.

**Figure 10:**
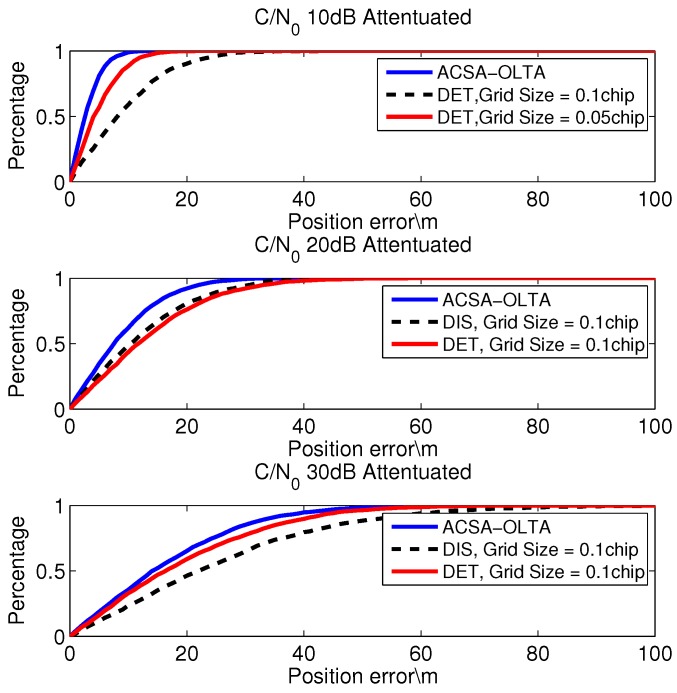
3-D position error cumulative distribution of open loop tracking.

In consideration of the fact that satellite signal power may be attenuated by sheltering, inappropriate antenna direction, *etc*. in an urban area, extra AGWN is added to change the $C/N_{0}$ of the received signal. Three tests are conducted in which the signal is attenuated by 10 dB, 20 dB and 30 dB. When it is 10 dB attenuated, for all the satellites, ACSA-OLTA computes the pseudo range with the discriminator based method. As a consequence, the three-Dimension (3-D) positioning error of DET with the fixed code search grid sizes of 0.1 chip and 0.05 chip is computed to compare with that of ACSA-OLTA. When $C/N_{0}$ is 20 dB and 30 dB attenuated, ACSA-OLTA changes the measurement extraction strategy and the 3-D positioning error of ACSA-OLTA is compared with DET and DIS in which the grid sizes are both 0.1 code chip. The 3-D position error cumulative distribution of the three tests is showed in Figure 10. The position error cumulative distribution is a statistic result of the position error. It calculates the percentage of position results with the 3-D error less than the given value in all positioning results. The expression of the position error cumulative distribution (PECD) is

(37) $$E_{PECD}\left(E_{g}\right)=\frac{1}{N_{p}}CNT\left(\left\{E_{n};E_{n}<E_{g},n=1,2,...,N_{p}\right\}\right)$$

where $E_{g}$ is the given position error and $E_{n}$ is the 3-D error of the nth position result. $N_{p}$ is the total number of the position results. CNT({x}) is the number of the elements in the set {x}.

When $C/N_{0}$ is 10 dB attenuated, the lowest $C/N_{0}$ of these satellites is 30 dBHz. For ASCA-OLTA and other open loop tracking methods with the fixed grid sizes, all of the satellites shown in Figure 9 can be detected and used for position. It is obvious that the EMLD applied by ASCA-OLTA brings higher positioning accuracy than the open loop methods which directly extract the measurement from the grid with the sizes of 0.1 chip and 0.05 chip. Since the $C/N_{0}$ is relatively high, the false detection hardly occurs and the uncertainty in the code search grid can be totally eliminated by the discriminator. As a result, the positioning accuracy of ASCA-OLTA is perfect. While, for the methods that directly extracting the code phase from the grid, the inherent uncertainty will probably distort the measurement accuracy so that the positioning accuracy is disturbed although the $C/N_{0}$ is high. As shown in the figure, the larger the grid size is, the worse the position accuracy is.

When $C/N_{0}$ is 20 dB attenuated, the lowest $C/N_{0}$ of these satellites is 20 dBHz and all of the satellites can still be detected. In this case, according to the signal $C/N_{0}$, the grid sizes of ACSA-OLTA for some of the satellites are narrowed down to the optimal grid size and the code phase measurement is directly extracted from the grid parameters. The results show that the positioning accuracy of the EMLD based open loop tracking method and the method directly extracting the measurement from the grid with the size of 0.1 chip are probably the same and both of them are poorer than ASCA-OLTA. Since the $C/N_{0}$ of the satellites ranges from 20 dBHz to 30 dBHz, both of the two methods have satellites with accuracy highly disturbed pseudo range measurements. While ASCA-OLTA can adaptively choose the optimal strategy and properly narrowing down the grid size based on the optimal ones so that the measurement accuracy can be greatly improved and the positioning accuracy is outstanding.

When $C/N_{0}$ is 30 dB attenuated, the signal power of all the satellites is really low and the positioning accuracy is degraded. During the test, all of the satellites except SV.25 can be detected all the time and the GDOP is still less than 2.0. In this case, the grid size of ACSA-OLTA is broadened to keep the correctness of the peak detection and it shows superiority over the open loop tracking method whose measurement is gained from the grid with the fixed size of 0.1 chip. The positioning error of the EMLD based method with the grid size of 0.1 chip is much larger than that of the ACSA-OLTA and it is even worse than the measurement directly gained from the grid. As a result, it is concluded that, when the $C/N_{0}$ is low, the noise will highly distort the accuracy of the discriminator based method. In addition, by appropriately broadening the detection grid size as the ACSA-OLTA does, the accuracy will be improved in the condition of low $C/N_{0}$.

### 4.3. Test in Urban Area

An experiment was conducted to further evaluate the performance of the proposed ACSA-OLTA in urban area according to the method in [28]. The same as the position accuracy test in Section 4.2, the experiment is carried out by off-line processing. The data utilized is GPS L1CA signal that is collected in Zhongguancun, Beijing, China where is a typical urban scenario. There are a lot of skyscrapers aside the road and the satellite signal is easily sheltered and attenuated so that it is a difficult environment for satellite navigation. The navigation message is gained by AGPS so that coherent integration length is not limited. The total duration of the test is about 30 min and the ephemeris gotten at the beginning is in effect all the time. The vehicular velocity varied from 0–40 km/h with random stops due to the traffic lights and the total distance traveled is about 5 km.

**Figure 11:**
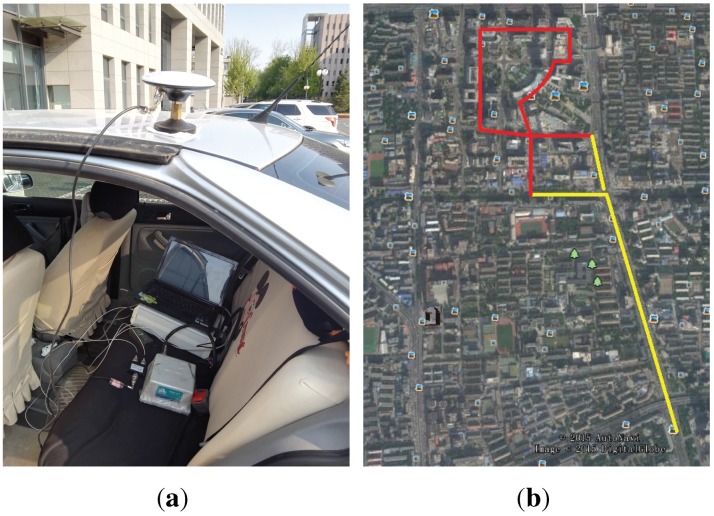
(**a**) Equipments of the test; (**b**) Trajectory of the test (Gained from Google Earth).

The test equipment is composed of a digital intermediate frequency signal collector which can down convert the satellite to intermediate frequency and store it in real time. Besides that, the SPAN-CPT Single Enclosure GNSS/INS Receiver is utilized to obtain the reference solution. It is an integrated system powered by NovAtel’s world class OEM6 technology which is comprised of Fiber Optic Gyros (FOG) and Micro Electrical Mechanical Systems (MEMS) accelerometers. It works in double difference mode using phase and Doppler measurements. The baseline separation (relative to a base station located on the Weigongcun) varied between 3–4 km. The reference solution accuracy in these conditions is summarized in Table 3. A power divider is utilized after the antenna and the two outputs are connected to the data collector and the SPAN-CPT receiver respectively to guarantee that the reference solutions and the collected data are correspondence. The equipments and trajectory of the test is showed in Figure 11.

**Table 3:** The reference solution accuracy.

Information	Accuracy
Position	0.1 m
Velocity	cm/level
Attitude	<1 deg

**Figure 12:**
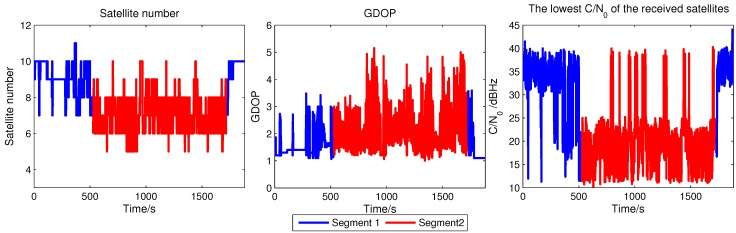
Variation of the satellite number, GDOP and $C/N_{0}$ in the test.

To facilitate the data analysis, the trajectory is divided into two segments which are marked by the yellow line and red line in Figure 11b. The yellow line is Segment 1 and the red line is Segment 2. They represents different operational scenarios that are considered and examined separately. The satellite number, GDOP and the lowest $C/N_{0}$ of all the satellite signal received during the test are drawn in Figure 12, where Segment 1 is drawn in blue and Segment 2 is in red.

Segment 1 is on a wide road where the satellite visibility is good and the operational conditions can be considered semi-open sky. In this area, the satellite is difficult to be sheltered and attenuated. In most time, the satellite number is larger than 8, the GDOP is smaller than 3 and the $C/N_{0}$ is above 30 dBHz. Segment 2 represents a very difficult scenario. As shown in the figure, although the sensitivity of open loop tracking is outstanding, there are sudden drops in the satellite number for the reason that the buildings can totally shelter the satellite signals propagating from one direction. The GDOP will fall down as well because of the poor constellation. There is also a long time when the $C/N_{0}$ is continuous low which is caused by the window plafond in the air. All of them will distort the positioning accuracy.

The primary objective of the experiments is to evaluate the code phase measurement accuracy of the proposed ACSA-OLTA and the traditional open loop tracking methods with fixed grid size and measurement calculation approach in urban area. Hence, the positioning algorithm is still LS which has no process of filter and is able to better indicate the measurement accuracy. Since the evaluation focuses on the effects of the code search grid size and measurement extraction method on the positioning accuracy of open loop tracking, other parameters such as coherent integration length and frequency search step are all identical among the methods tested. The main parameters of ACSA-OLTA are listed in Table 4. Besides that, the $C/N_{0}$ for ACSA-OLTA to adjust the correlation space is accurate to 1dB. Since the $C/N_{0}$ of NovAtel receiver is estimated from the correlator whose code phase is best aligned with the incoming signal, which is the same as that estimated in the process of ACSA-OLTA, the test results by using the two sources of $C/N_{0}$ will be almost the same. Thus, the $C/N_{0}$ of NovAtel receiver is utilized as reference for simplicity. The parameters of the traditional methods with fixed grid sizes and measurement calculation approaches considered for comparison are also listed in Table 4. The abbreviations of DET and DIS represent the open loop tracking methods with the code phase measurement computed from the EMLD and directly extracted from the grid respectively.

**Table 4:** The parameters of the methods tested.

Method	Grid Size	Frequency Step	Integration Length	Positioning Rate
ACSA-OLTA	Adaptively adjusted based on $C/N_{0}$ as Figure 5	5 Hz	300 ms	1 Hz
DIS	0.1 chip	5 Hz	300 ms	1 Hz
DET	0.2 chip	5 Hz	300 ms	1 Hz
	0.1 chip	5 Hz	300 ms	1 Hz
	0.05 chip	5 Hz	300 ms	1 Hz
	0.02 chip	5 Hz	300 ms	1 Hz

**Figure 13:**
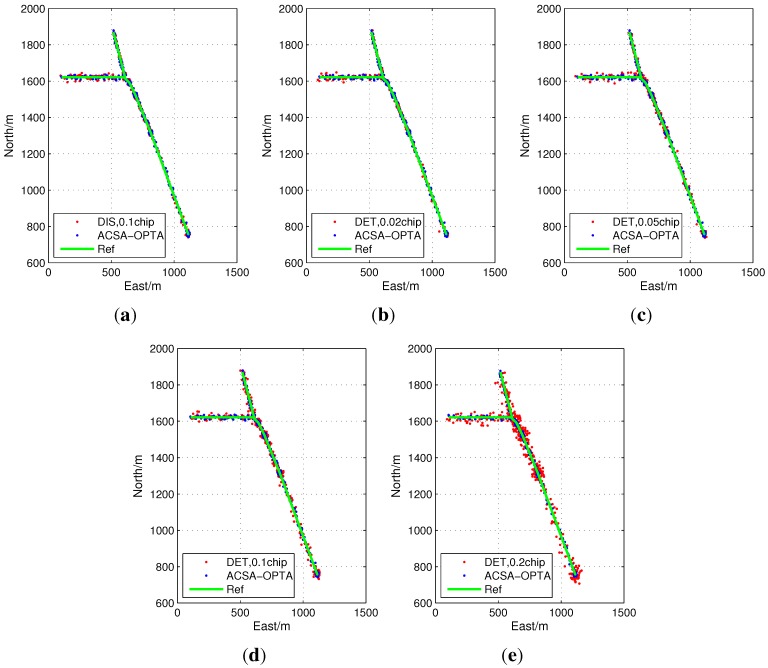
Positioning results of open loop tracking in Segment 1.

Figure 13 illustrates the positioning results of the proposed ACSA-OLTA together with DIS with the grid size of 0.1 chip, DET with the grid sizes of 0.2 chip, 0.1 chip, 0.05 chip and 0.02 chip in Segment 1. The solutions of DET with the grid sizes of 0.1 chip and 0.2 chip shows large errors during the test. Narrowing down grid sizes shows evident improvements relative to those with larger grid sizes. As the $C/N_{0}$ is high, the detection performance of open loop tracking system is perfect. In this scenario, for DET, the most accuracy peak is easily detected and the measurement will be improved when the grid size is small. Whereas the large uncertainty in wide grid will deteriorate the accuracy. In addition, DIS provides better performance and it is similar to DET with the grid size of 0.02 and 0.05 chip. All of the results are coincide with the analysis in Section 3. Finally, the trajectory obtained by ACSA-OLTA, in which most of the code phase measurements are calculated by the EMLD with the grid size of 0.1 chip, shows perfect positioning accuracy with only small disagreements with the reference trajectory.

**Figure 14:**
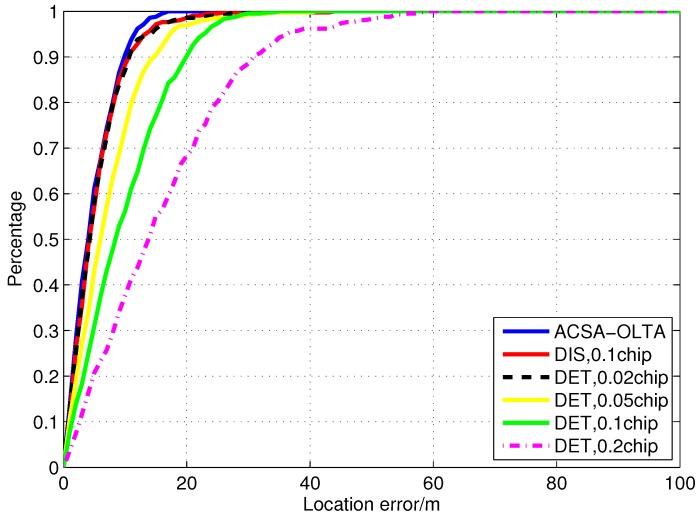
Position error cumulative distribution of the positioning results in Segment 1.

In order to further clarify the contribution of the proposed ACSA-OPTA, the position error in the segment is evaluated by the position error cumulative distribution expressed in Equation (37). The result is drawn in Figure 14. As can be seen from the figure, the accuracy of DET with the grid size of 0.02 chip and DIS with the grid size of 0.1 chip is close and 90% position results have the error less than 10 m. DIS is preferable due to the low computational costs and reduction of the correlators. When the grid size is 0.05 chip, the accuracy of DET is a little degraded and 80% position errors are smaller than 10 m. However, when the grid size of DET extends to 0.1 chip, 40% positioning results have error larger than 10 m. For DET with the grid size of 0.2 chip, the maximum error reaches about 40 m and only 40% positioning results have error less than 10 m. ACSA-OPTA is better than all the other methods tested because there is still some sudden power reduction during the segment. ACSA-OPTA can adaptively adjust the grid size and measurement extraction method to keep accuracy.

**Figure 15:**
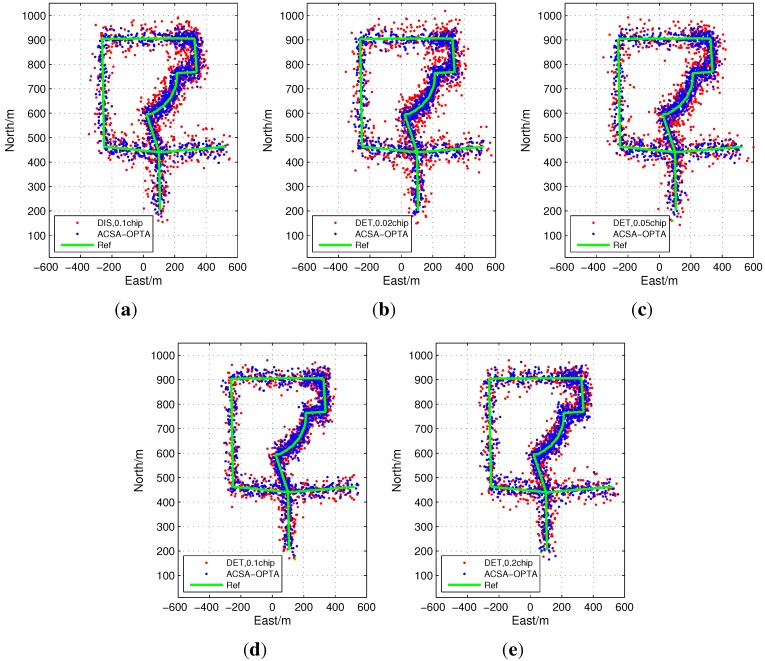
Positioning results of open loop tracking in Segment 2.

The trajectories obtained with the open loop tracking with different configurations for Segment 2 are shown in Figure 15. The position accuracy of DET with small grid sizes is highly degraded. Owing to the poor detection performance caused by the low $C/N_{0}$, false peak detection frequently occurs which will extremely distort the position results especially when the grid size is small. The maximum error reaches about 80 m. For DIS, the position error is also larger since the huge noise in the signal destroys the estimation results. As the grid size is broadened to 0.1 chip for DET, the positioning performance becomes better. However, when the grid size of DET is 0.2 chip, the error is a little larger due to the incremental uncertainty within the grid. ACSA-OPTA shows remarkable improvements relative to the other configurations. As $C/N_{0}$ is varying intensely, the ACSA-OPTA can adaptively adjust the grid size to the optimal one so that it can enhance the measurement accuracy in all cases.

**Figure 16:**
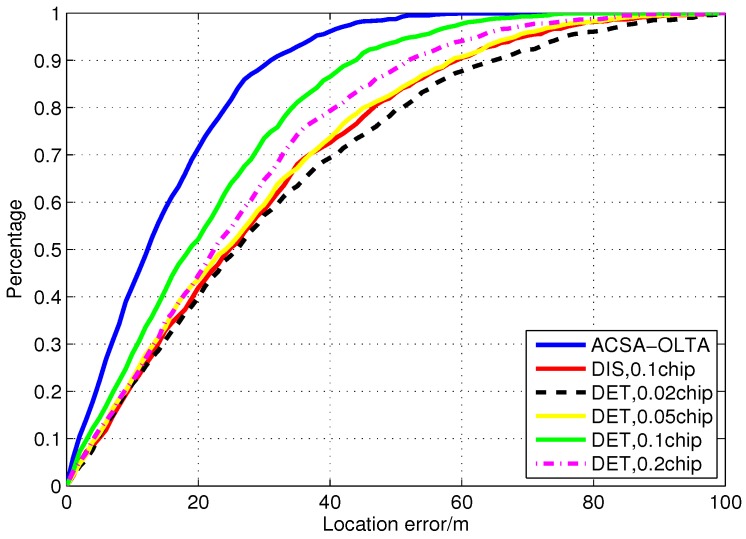
Position error cumulative distribution of the positioning results in Segment 2.

Figure 16 illustrates the position error cumulative distribution of the results in Segment 2. It shows that the performance of ACSA-OLTA is outstanding compared with the traditional open loop tracking methods whose configurations of grid sizes and measurement calculation method are fixed. 70% of the positioning results have error smaller than 20 m and less than 10% positioning results have errors larger than 40 m. In addition, the positioning errors of DET with the grid size of 0.02 chip and 0.05 chip and DIS with the size of 0.1 chip are extremely increased. More than 30% position results have error larger than 40 m. On the other hand, large numbers of positioning results computed from DET with the grid size of 0.1 chip have the errors in the range from 15 m to 40 m due to the fact that it can avoid the false detection to some extent but the uncertainty will distort the accuracy as well. Continuously enlarging the grid size to 0.2 chip, the performance of DET is degraded as well and more of the position results have the error larger than 20 m. Although it can eliminate the false peak detection in most time, the uncertainty in the grid is really large so that the accuracy is also distorted.

## 5. Conclusions

This work looks at the pseudo range measurement of open loop tracking for GNSS and proposes the Adaptive Correlation Space Adjusted Open Loop Tracking Approach (ACSA-OLTA) to improve the measurement accuracy of open loop tracking for vehicle positioning in urban area. A novel expression to obtain the code search grid size which can bring the best measuring accuracy, named Equivalent Weighed Pseudo Range Error is presented in Section 3.1. Using the new expression, the optimal code search grid sizes for different $C/N_{0}$ are computed. Following a thorough analysis, the accuracy of the code phase measurement calculated from the early-minus-late discriminator and that directly extracted from the grid is evaluated for different grid sizes when $C/N_{0}$ varies. The computational costs are also assessed. Based on the analysis results, the measurement calculation strategy is developed. ACSA-OLTA adaptively adjusts the correlation space to the optimal code search grid size and switches the measurement calculation methods as the developed strategy according to $C/N_{0}$.

With the help of the Monte Carlo simulation, the mean code phase measuring errors for different $C/N_{0}$ and grid sizes are evaluated. The results verify that the proposed EWPRE can indicate the measuring error level for open loop tracking with different grid sizes. The optimal code search grid size obtained according to EWPRE for the corresponding $C/N_{0}$ is valid. Data was collected in the campus of Beijing Institute of Technology and Zhongguancun, Beijing and processed using various configurations. For the data analyzed herein, based on the positioning results computed with the Least Square method, which is directly related to the code phase measurement accuracy, the main conclusions are as follows:

- In open air area where the $C/N_{0}$ of most satellites are larger than 30 dBHz, the errors of most positioning results calculated from the code phase measurements that are gained from the EMLD is smaller than 10 m. It outperforms those directly extracted from the grid with the sizes of 0.05 chip, 0.1 chip and 0.2 chip and it is similar to that with the grid size of 0.02 chip. The results validate the analysis in Section 3.2 that the early-minus-late discriminator can keep the measurement accuracy but reduce the correlator utilized when $C/N_{0}$ is high. It also indicates that narrowing down grid size can help to improve the accuracy in this case which is the same as that EWPRE shows.
- In urban canyon area where the $C/N_{0}$ of a number of satellites is lower than 20 dBHz, the performance of the EMLD based measurement calculation method is greatly degraded. At the same time, the accuracy of the measurements directly extracted from the grids with size of 0.02 chip and 0.05 chip are also deteriorated. On the contrary, the accuracy of the measurements directly extracted from grids with larger grid sizes is better. When the grid sizes are 0.1 chip and 0.2 chip, the positioning errors are much less than those with smaller grid sizes. It verifies that properly enlarging the search grid size can improve the measurement accuracy when $C/N_{0}$ is low as the EWPRE shows in Section 3.1.
- In both the two scenarios, ACSA-OLTA can greatly improve the measurement accuracy compared with the traditional open loop tracking method with fixed search grid sizes and measurement calculation methods. The results of the test in the paper show that when $C/N_{0}$ is high, the EMLD is utilized and 90% positioning results have the error smaller than 10 m. When $C/N_{0}$ is low, the search grid size is adjusted and 70% positioning results have the error smaller than 20 m. The results also validate that the grid sizes gained from EWPRE are preferable.

Based on these results, ACSA-OLTA does improve the measurement accuracy of open loop tracking and enhance the positioning performance of the open loop tracking based GNSS receiver for vehicular application in urban areas.

## Appendix

According to Equation (33), if the discriminator is perfectly normalized and the front-end bandwidth is infinite, the variance of the discriminator output is

(A1) $$\sigma_{EML}^{2}=\left(Var\left[E_{I}^{2}-L_{I}^{2}\right]+Var\left[E_{Q}^{2}-L_{Q}^{2}\right]\right)/k_{d}^{2}$$

where Var[x] is the variance of *x*. $k_{d}$ is the gain of the discriminator which is

(A2) $$\frac{\partial }{\partial \tau }\left[\left(E_{I}^{2}+E_{Q}^{2}\right)-\left(L_{I}^{2}+L_{Q}^{2}\right)\right]=4P\left(1-\tau_{b}\right)$$

Taking $Var[E_{I}^{2}-L_{I}^{2}]$ as an example, the variance is

(A3) $$Var\left[E_{I}^{2}-L_{I}^{2}\right]=Var\left[E_{I}^{2}\right]+Var\left[L_{I}^{2}\right]-2cov\left(E_{I}^{2},L_{I}^{2}\right)$$

where

$$\begin{array}{cc} & Var\left[E_{I}^{2}\right]=4PR^{2}\left(\tau -k\tau_{b}-\tau_{b}\right)cos^{2}\Delta \varphi \cdot E\left[n_{I,E}^{2}\right]+2{\left(E\left[n_{I,E}\right]\right)}^{2}\\ & Var\left[L_{I}^{2}\right]=4PR^{2}\left(\tau -k\tau_{b}+\tau_{b}\right)cos^{2}\Delta \varphi \cdot E\left[n_{I,L}^{2}\right]+2{\left(E\left[n_{I,L}\right]\right)}^{2}\\ & cov\left(E_{I}^{2},L_{I}^{2}\right)=4PR^{2}\left(2\tau_{b}\right)cos^{2}\Delta \varphi \cdot E\left[n_{I,E}n_{I,L}\right]+E\left[n_{I,E}^{2}n_{I,L}^{2}\right]-E\left[n_{I,E}^{2}\right]E\left[n_{I,L}^{2}\right] \end{array}$$

cov(x,y) is the covariance between *x* and *y*. According to Equations (2) and (3), the noise components of the in-phase correlation outputs $E_{I}$ and $L_{I}$ are as below. The noise components of quadrature correlation outputs are more or less the same and the only difference is the local carrier multiplied is sine wave instead of the cosine wave of the in-phase branches.

(A4) $$n_{I,E}=\frac{1}{N_{c}}\sum_{i=0}^{N_{c}-1}n\left(t_{i}\right)c\left(t_{i}-k\tau_{b}-\tau_{b}\right)cos\left(2\pi \left(f_{IF}+f_{k}\right)t_{i}+\varphi \right)$$

(A5) $$n_{I,L}=\frac{1}{N_{c}}\sum_{i=0}^{N_{c}-1}n\left(t_{i}\right)c\left(t_{i}-k\tau_{b}+\tau_{b}\right)cos\left(2\pi \left(f_{IF}+f_{k}\right)t_{i}+\varphi \right)$$

The product of $n_{I,E}$ and $n_{I,L}$ is

(A6) $$\begin{array}{cc} n_{I,E}n_{I,L}= & \frac{1}{N_{c}^{2}}\sum_{i=0}^{N_{c}-1}\sum_{j=0}^{N_{c}-1}[n\left(t_{i}\right)c\left(t_{i}-k\tau_{b}-\tau_{b}\right)n\left(t_{j}\right)c\left(t_{j}-k\tau_{b}+\tau_{b}\right)\\ & \times cos\left(2\pi \left(f_{IF}+f_{k}\right)t_{i}+\varphi \right)cos\left(2\pi \left(f_{IF}+f_{k}\right)t_{j}+\varphi \right)] \end{array}$$

The mean value of $n_{I,E}^{2}n_{I,L}^{2}$ is

(A7) $$\begin{array}{cc} & E\left[n_{I,E}^{2}n_{I,L}^{2}\right]=E[(\frac{1}{N_{c}^{2}}\sum_{i=0}^{N_{c}-1}\sum_{j=0}^{N_{c}-1}n\left(t_{i}\right)c\left(t_{i}-k\tau_{b}-\tau_{b}\right)n\left(t_{j}\right)c\left(t_{j}-k\tau_{b}+\tau_{b}\right)\\ & \times cos\left(2\pi \left(f_{IF}+f_{k}\right)t_{i}+\varphi \right)cos\left(2\pi \left(f_{IF}+f_{k}\right)t_{j}+\varphi \right){)}^{2}]\\ = & E[\frac{1}{N_{c}^{2}}\sum_{i=0}^{N_{c}-1}\sum_{j=0}^{N_{c}-1}[n^{2}\left(t_{i}\right)c^{2}\left(t_{i}-k\tau_{b}-\tau_{b}\right)n^{2}\left(t_{j}\right)c^{2}\left(t_{j}-k\tau_{b}+\tau_{b}\right)\\ & \times cos^{2}\left(2\pi \left(f_{IF}+f_{k}\right)t_{i}+\varphi \right)cos^{2}\left(2\pi \left(f_{IF}+f_{k}\right)t_{j}+\varphi \right)]]\\ & +2E[\frac{1}{N_{c}^{2}}\sum_{i=0}^{N_{c}-1}\sum_{j=0}^{N_{c}-1}[n^{2}\left(t_{i}\right)c\left(t_{i}-k\tau_{b}-\tau_{b}\right)c\left(t_{i}-k\tau_{b}+\tau_{b}\right)n^{2}\left(t_{j}\right)c\left(t_{j}-k\tau_{b}-\tau_{b}\right)c\left(t_{j}-k\tau_{b}+\tau_{b}\right)\\ & \times cos^{2}\left(2\pi \left(f_{IF}+f_{k}\right)t_{i}+\varphi \right)cos^{2}\left(2\pi \left(f_{IF}+f_{k}\right)t_{j}+\varphi \right)]]\\ = & \left(1+2R^{2}\left(2\tau_{b}\right)\right)\cdot N_{0}/2T_{c}\cdot N_{0}/2T_{c} \end{array}$$

Supposing that τ is equal to $k\tau_{b}$ which is the best case that the grid parameter of the peak is that of the incoming signal, $Var[E_{I}^{2}-L_{I}^{2}]$ and $Var[E_{Q}^{2}-L_{Q}^{2}]$ are

(A8) $$Var\left[E_{I}^{2}-L_{I}^{2}\right]=8PR\left(\tau_{b}\right)cos^{2}\Delta \varphi \left(1-R\left(2\tau_{b}\right)\right)\cdot N_{0}/2T_{c}+4\left(1-R^{2}\left(2\tau_{b}\right)\right)\cdot N_{0}/2T_{c}\cdot N_{0}/2T_{c}$$

(A9) $$Var\left[E_{Q}^{2}-L_{Q}^{2}\right]=8PR\left(\tau_{b}\right)sin^{2}\Delta \varphi \left(1-R\left(2\tau_{b}\right)\right)\cdot N_{0}/2T_{c}+4\left(1-R^{2}\left(2\tau_{b}\right)\right)\cdot N_{0}/2T_{c}\cdot N_{0}/2T_{c}$$

Hence,

(A10) $$\sigma_{EML}^{2}=\frac{\tau_{b}}{2\cdot C/N_{0}\cdot T_{c}}\left(1+\frac{1}{C/N_{0}\cdot T_{c}\left(1-\tau_{b}\right)}\right)$$
